# Teaching Intuitive Eating and Acceptance and Commitment Therapy Skills Via a Web-Based Intervention: A Pilot Single-Arm Intervention Study

**DOI:** 10.2196/resprot.5861

**Published:** 2016-10-14

**Authors:** Sara Boucher, Olivia Edwards, Andrew Gray, Shyamala Nada-Raja, Jason Lillis, Tracy L Tylka, Caroline C Horwath

**Affiliations:** ^1^ Department of Human Nutrition University of Otago Dunedin New Zealand; ^2^ Department of Preventive and Social Medicine University of Otago Dunedin New Zealand; ^3^ Department of Psychiatry and Human Behavior Brown University Medical School Providence, RI United States; ^4^ Department of Psychology The Ohio State University Columbus, OH United States

**Keywords:** Web-based intervention, overweight, obesity, prevention, middle-aged women, BMI, intuitive eating, acceptance and commitment therapy

## Abstract

**Background:**

Middle-aged women are at risk of weight gain and associated comorbidities. Deliberate restriction of food intake (dieting) produces short-term weight loss but is largely unsuccessful for long-term weight management. Two promising approaches for the prevention of weight gain are intuitive eating (ie, eating in accordance with hunger and satiety signals) and the development of greater psychological flexibility (ie, the aim of acceptance and commitment therapy [ACT]).

**Objectives:**

This pilot study investigated the usage, acceptability, and feasibility of “Mind, Body, Food,” a Web-based weight gain prevention intervention prototype that teaches intuitive eating and psychological flexibility skills.

**Methods:**

Participants were 40 overweight women (mean age 44.8 [standard deviation, SD, 3.06] years, mean body mass index [BMI] 32.9 [SD 6.01] kg/m^2^, mean Intuitive Eating Scale [IES-1] total score 53.4 [SD 7.46], classified as below average) who were recruited from the general population in Dunedin, New Zealand. Module completion and study site metrics were assessed using Google Analytics. Use of an online self-monitoring tool was determined by entries saved to a secure online database. Intervention acceptability was assessed postintervention. BMI, intuitive eating, binge eating, psychological flexibility, and general mental and physical health were assessed pre- and postintervention and 3-months postintervention.

**Results:**

Of the 40 women enrolled in the study, 12 (30%) completed all 12 modules (median 7.5 [interquartile range, IQR, 2-12] modules) and 4 (10%) used the self-monitoring tool for all 14 weeks of the intervention period (median 3 [IQR 1-9] weeks). Among 26 women who completed postintervention assessments, most women rated “Mind, Body, Food” as useful (20/26, 77%), easy to use (17/25, 68%) and liked the intervention (22/25, 88%). From pre- to postintervention, there were statistically significant within-group increases in intuitive eating (IES-2 total score *P*<.001; all IES-2 subscale scores: *P* ≤.01), psychological flexibility (*P*=.01), and general mental health (*P*<.001) as well as significant decreases in binge eating (*P*=.01). At the 3-month follow-up, IES-2 improvements were maintained, and there were further improvements in binge eating (*P*<.001) and general mental health (*P*=.03), and a marginal yet nonsignificant tendency for further improvement in psychological flexibility (*P*=.06). There were no significant within-group changes in BMI from pre- to postintervention and postintervention to 3-month follow-up (*P*=.46 and *P*=.93, respectively).

**Conclusions:**

The “Mind, Body, Food” prototype Web-based intervention is appealing to middle-aged women and may be a useful tool to help women learn intuitive eating and ACT skills, reduce binge eating, and maintain weight over 3 months. Further work to improve the user experience and engagement is required before testing the online intervention in a randomized controlled trial.

## Introduction

Despite the high prevalence of reported attempts at weight control, obesity rates worldwide continue to increase [1-3]. Since obesity is associated with many adverse health consequences [4], and weight loss interventions have shown modest long-term success [5-7], there is an increasing focus on the prevention of weight gain [8,9]. Among middle-aged women (40-50 years), weight gains of 0.5 to 0.7 kg per year have been observed [10-12], and there is evidence that the greatest risk for weight gain is among women who are already overweight or obese [10]. Around menopause, weight gain is associated with increases in cardiovascular risk factors such as blood pressure, total cholesterol, low-density lipoprotein cholesterol, triglycerides, and fasting insulin [12]. For these reasons, effective weight gain prevention has been identified as an important goal for premenopausal women [12], because even among middle-aged women who report following healthy weight control behaviors (eg, decreased food quantity, exercise, reducing fat intake), weight gain is observed [1].

Intuitive eating is a nondieting, adaptive approach to eating behavior characterized by eating for physical rather than emotional reasons, relying on internal hunger and satiety cues, unconditional permission to eat when hungry and what food is desired, and choosing nutritious foods to help one’s body function well [13-15]. Although intuitive eating shares similarities with mindful eating, there are differences. Mindful eating involves paying attention, without judgment, to the eating experience and to hunger/satiety cues and eating attentively without distraction [16]. However, intuitive eating also takes nutrition into consideration when choosing foods to enhance/support body function and involves giving oneself unconditional permission to eat not only when hungry but those foods one truly feels like eating [13-15]. Cross-sectional research has shown intuitive eating to be positively related to body appreciation, self-esteem, and satisfaction with life and inversely related to eating disorder symptomatology, poor interoceptive awareness, body surveillance, body shame, body mass index (BMI), and internalization of media appearance ideals [14]. Intuitive eating was inversely associated with BMI in a nationwide sample of middle-aged women [17] and intuitive eating interventions have lowered cholesterol levels, blood pressure, disordered eating, body dissatisfaction, and depression and improved diet quality, physical activity, stress management, and self-esteem [18,19]. Compared to diet group participants, participants who practice intuitive eating have been shown to maintain improvements in metabolic function (eg, blood pressure, blood lipids) even in the absence of weight loss, whereas little improvement was sustained in diet group participants [19]. A recent review of 20 studies evaluating intuitive eating interventions [18] reported reductions in weight in 6 studies, weight maintenance in 8 studies, and mixed results in 2 studies.

Long-term weight management is enhanced by effective emotion regulation skills [20,21]. A growing body of evidence supports the effectiveness of acceptance and commitment therapy (ACT) in the management of obesity [22-27]. ACT-based interventions focus on improving psychological flexibility, which is the ability to remain mindfully aware and accepting of one’s experience in the present moment (eg, thoughts, feelings, bodily sensations) while also clarifying one’s values and choosing to engage in behavior that is consistent with those values [28]. ACT-based skills do not teach control or avoidance of eating triggers but rather develop skills that create a different relationship with these triggers: allowing individuals to be present without acting on the triggers (ie, acceptance). Acceptance-based interventions (ie, teaching skills such as values clarity and behavioral commitment, awareness of decision-making processes, and distress tolerance) have been shown to increase psychological flexibility and improve weight control, particularly for those who tend to eat in response to emotional and environmental triggers [29]. In randomized controlled trials (RCTs) among adults attempting weight loss, brief (ie, 6-8 hours) ACT-based interventions have been reported to be more effective for weight loss compared to no-treatment [25,27]. Tapper’s [27] findings suggest that the effect of the intervention on BMI was largely brought about by reductions in binge eating, while Lillis [25] reported that weight-specific acceptance and psychological flexibility significantly mediated BMI outcomes. Recently, Sairanen et al [30] reported that among overweight adults, the ability to recognize and accept aversive internal experiences (without reacting to them) was positively associated with eating for physical rather than emotional reasons.

Intuitive eating and ACT appear to be particularly well suited for integration, and to our knowledge, this is the first study to test them when used together. Both approaches focus on fostering a mindful, accepting, and open stance to one’s experiences. Intuitive eating teaches individuals to be more mindfully aware of and follow the body’s hunger and satiety cues, while ACT teaches individuals how to cope more effectively with negative and unwanted cognitive and emotional cues through awareness and acceptance. ACT skills may complement intuitive eating by helping to decouple eating behavior from emotional cues and external triggers, allowing for hunger and satiety cues to play a stronger role in regulating eating behavior.

Web-based interventions have the ability to have a major public health impact. They can present complex health information in simple formats (eg, video, graphic, audio) [31], overcome time and travel barriers of face-to-face interventions [32], reach a large audience [33], reduce stigma related to being overweight or obese [34], and facilitate weight management [35]. ACT interventions have been adapted for Web-based delivery and have been shown to be effective in teaching ACT-based skills to manage conditions such as tinnitus [36], chronic pain [37], work-related stress [38], and multiple behavioral health risks [39]; however, there are no published studies testing Web-based ACT for eating behavior interventions. Although a small number of studies have investigated Web-based healthy eating interventions based on nondiet and size acceptance approaches among young adults [40,41], no published study to date has, to our knowledge, designed and evaluated an online intuitive eating intervention. Integrating ACT and intuitive eating is a novel approach to changing eating behavior that when delivered online would provide an accessible weight management intervention grounded in evidence-based theory.

We developed an online intuitive eating intervention called “Mind, Body, Food,” based on the intuitive eating principles set forth by Tribole and Resch [15] and incorporating ACT-based skills. The aim of “Mind, Body, Food” was to teach middle-aged women intuitive eating skills facilitated via acquiring ACT-based skills. It was theorized that increased psychological flexibility resulting from the acquired ACT-based skills will further enable women to eat more intuitively by helping them to handle feelings, thoughts, urges, or cravings that can trigger eating when not physically hungry. The aim of this pilot study was to design an evidence-based prototype online intuitive eating intervention and investigate its acceptability, feasibility, and usage among middle-aged women.

## Methods

### Research Design and Setting

For this prospective single-arm pilot intervention study, participants were recruited from the community in Dunedin, New Zealand, and assessed preintervention, postintervention, and at 3-month follow-up. They accessed the intuitive eating intervention directly via the Internet. The University of Otago Human Ethics Committee granted ethical approval (reference code: H13/057). The Ngāi Tahu Research Consultation Committee approved the research methods as being consistent with the needs of the Ngāi Tahu iwi (South Island Māori, the indigenous population of New Zealand).

### Participants and Recruitment

Women aged 40 to 50 years (inclusive) were eligible to participate in the study if they were able to communicate in English, able to engage in gentle physical activity (ie, walk at a leisurely pace for 10 minutes or more), premenopausal (ie, having a menstrual period in the preceding three months or currently being on any form of hormonal contraceptives that stopped menstruation [42]), and regularly accessed the Internet and email at least three days per week. To target women with the greatest potential to benefit from training in intuitive eating, we further restricted inclusion to women with below average Intuitive Eating Scale scores (IES) [13] (summed total IES score less than 65, based on nationwide survey data for overweight NZ women [17]) and with a BMI of at least 26.5 kg/m^2^ (calculated using measured height and weight). Women were excluded if they were pregnant or lactating, taking hormone replacement therapy, or a current smoker or if they had a history of hysterectomy, oophorectomy, diabetes, cancer, cardiovascular disease, or eating disorder. Additionally, women were excluded if they currently were taking a medication that may affect their appetite or were enrolled in any weight loss program.

To recruit women from a diverse range of socioeconomic and ethnic backgrounds, recruitment materials were circulated through local health promotion and social service networks, low socioeconomic neighborhoods, and organizations servicing Pacific and Māori populations. Recruitment materials were framed in the context of encouraging women interested in learning intuitive eating skills for long-term weight management. The study was also promoted in a local newspaper article.

### Sample Size

We aimed for a sample size of 40 participants at the 3-month follow-up visit in order to investigate acceptability and usability as well as providing sufficiently precise estimates of outcome measure variability, correlations between repeated measures, and retention rates. The sample size was not determined according to statistical principles but was the number judged to be suitable for achieving the study objectives. The recruitment target was set at 58 women to account for an estimated 30% attrition rate, based on previous research [43].

### “Mind, Body, Food” Intervention

**Figure 1:**
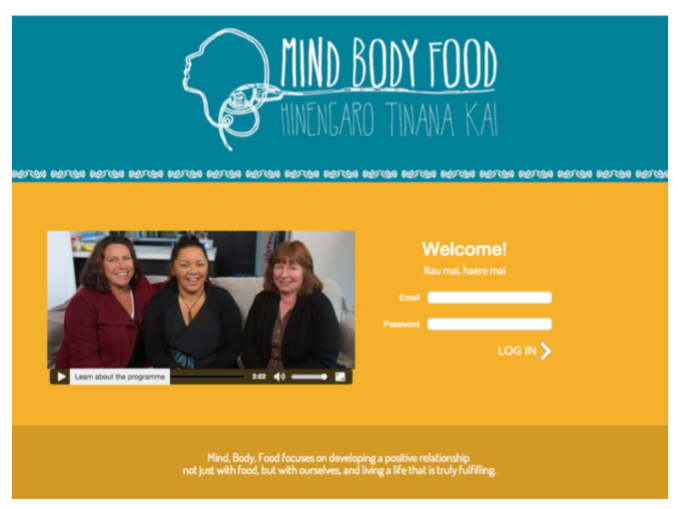
"Mind, Body, Food" log-in page.

The 12-module self-guided “Mind, Body, Food” intervention was developed through an iterative process involving extensive input from end-users. The content was guided by evidence-based research in the field [25,27] and the researchers’ own experience teaching intuitive eating (CCH, SB, TLT) and ACT skills (CCH, JL) face-to-face. A paper-based intervention prototype was pretested in focus groups and interviews with members of the target audience, followed by pretesting of a Microsoft PowerPoint-based prototype of the intervention. Following this, expert review of the intervention material, with a particular focus on intuitive eating (TT) and ACT (JL) content, was undertaken to ensure clarity and fidelity to the underpinning theoretical basis for the intervention. “Mind, Body, Food” content was then translated by a professional Web developer into a Web-based prototype (Figures 1 through 5), which was not compatible with mobile browsers. The Web-based prototype underwent usability and heuristic testing before the current study.

“Mind, Body, Food” consists of 12 modules, each covering skills related to intuitive eating and taking 15 to 20 minutes to complete. Table 1 presents “Mind, Body, Food” module titles and key skills and activities delivered. In each module, the key skills and their rationale were introduced in a 3- to 5-minute video featuring a discussion among three women (one dieter and two women who had learned intuitive eating). Each video commenced with discussion of a challenge related to eating (eg, recognizing physical hunger) before introducing the new skills and a discussion of the women’s experience of practicing the skills. Each module followed with a guided audio activity and typed activity. Guided experiential audio activities were used to deliver training in recognition of hunger and fullness signals and many of ACT’s core skills (eg, awareness and acceptance of thoughts and feelings; “surfing” urges to eat when not physically hungry [ie, acceptance]; visualizing placing thoughts on leaves floating in a stream [ie, ACT’s cognitive defusion skill]). Typed activities encouraged participants to reflect on how practicing the skills would make a difference to their life (ie, clarification of values). At the end of each module, users were encouraged to practice intuitive eating skills every day. Starting in Module 2, use of the Eating Awareness Tracker (EAT), a self-monitoring tool for recording nonjudgmental observations of eating-related experiences, was encouraged, and a library of additional resources was provided. The EAT enabled participants to monitor hunger, fullness, and mindfulness ratings during eating, each using 0 to 10 scales. Beginning in Module 1, the EAT was presented as an essential part of the intervention. Participants were encouraged to log into “Mind, Body, Food” to use the EAT as often as possible after meals or snacks and were provided an opportunity to practice using the EAT during Modules 2-11 by recording the most recent eating experience. At the end of each module, participants could choose to receive, via email or short message service (SMS), up to three daily prompts to use the EAT.

**Table 1:** “Mind, Body, Food” modules (including the te reo Māori titles) and key skills taught in each module.

Module	Title	Key skills	Activities
1	Ditch the Diets / Whakarerea te whakapuango	Giving self permission to eat wide range of foods. Guiding food choices based on what feels enjoyable and satisfying.	Practice before each snack/meal asking self, “It’s okay to have this, but will I truly enjoy this and feel satisfied?” Typed and audio activities: reflection on past experience of dieting, or labeling foods “forbidden.”
2	Tuning into Hunger / E rongo ana i te hikai	Recognizing what physical hunger feels like. Before eating, rating physical hunger on a scale 0 (absolutely empty) to 10 (sick from overeating). Initiating eating when hunger is rated 3 or 4.	Guided audio activity: scan of mouth, throat, and stomach to recognize physical signals of hunger. Typed activities: reflect on sensations of hunger (or lack of) before typical eating experiences, and reflect on how life would be different if body signals were listened to more often before eating.
3	Am I Full? Kua puta a pito?	Recognizing what fullness feels like and knowing when to take the last bite. While eating, rating fullness on a scale of 0 (absolutely empty) to 10 (sick from overeating). Finishing eating when fullness is rated 6 or 7.	Guided audio activity: awareness of stomach sensations to recognize physical signals of fullness. Typed activities: reflect on sensations of fullness after eating, and reflect on how life would be different if body signals were trusted to guide how much to eat.
4	One Bite at a Time / Kei ia ngau; ka ngau, a, ka ngau	Eating with nonjudgmental awareness (ie, mindfulness). Rating mindful eating on a scale from 0 (mindless eating) to 10 (mindful eating). Mindfully eating in 7 to 10 rating range.	Guided audio activity: practice mindfully eating a piece of dried fruit. Typed activity: reflect on usual level of mindfulness when eating and how the experience of eating would differ if more attention was paid to the food and how food affects the body.
5	Coping with Cravings / Whakataha atu te wararwara	Coping with urges to eat when not physically hungry (ie, acceptance).	Guided ACT audio activity: “urge surfing” to cope with a craving to eat when not physically hungry. Typed activities: reflect on common triggers to eat when not physically hungry and how life would be different if response to cravings was asking, “Am I physically hungry?” and “Is this food really what I feel like in this moment?”
6	Emotional Eating / Ka kai ki te whakarata i te mānuka	Identifying emotional triggers to eat (ie, acceptance). Coping with emotions without using food.	Guided ACT audio activity: practice making space for emotions (ie, not struggling to change them or get rid of them). Typed activities: reflect on common triggers to eat emotionally and how life would be different if allowing uncomfortable feelings to be present and not eating to change feelings. Ask, “Is emotional eating in line with what matters most in life?”
7	Every Body Deserves Respect / He mana tō ia tinana	Shifting focus from body appearance to appreciating body functions.	Guided audio activity: body scan with appreciation of body functions. Typed activities: describe self with nonjudgmental (ie, neutral or positive) words, and reflect on how life would be different if focus shifted from changing body’s appearance to appreciating body’s functions.
8	Dealing with Pressures to Diet / Whaihangatia ngā pēhanga o te whakapuako	Handling pressures to diet or engage in “fat talk.”	Audio activity: review of diet cycle, encourage body appreciation and reflection on positive experiences with intuitive eating to reinforce motivation to eat intuitively. Typed activities: reflect on positive changes made since beginning “Mind, Body, Food,” and identify responses to pressures to diet and “fat talk.”
9	Taming the Inner Critic / Whakarata te kaiwhakatāwai o roto	Distancing self from negative thoughts (ie, cognitive defusion).	Guided ACT audio activity: visualize placing unhelpful thoughts on leaves in a stream. Typed activities: identify techniques to “unhook” from unhelpful thoughts, and reflect on how life would differ if not attached to unhelpful thoughts.
10	Get Active Your Way / Kei a koe te tikanga korikori	Engaging in enjoyable physical activities every day.	Guided audio activity: Walking mindfully (ie, tuning into bodily sensations and surroundings while walking). Typed activities: reflect on benefits of physical activity and how to include physical activity in daily routine.
11	Fine Tuning Food Choices / Āta whiria te kai	Selecting healthier food options without feeling deprived.	Guided audio activity: mindful food shopping. Typed activity: identify enjoyable ways to eat more fruits and vegetables and choose lower fat foods without feeling deprived.
12	Staying on Track / Kia mau, kia ū	Staying motivated to maintain changes and recovering from “slips.”	Guided audio and typed activities: reflect on positive changes made since starting “Mind, Body, Food,” and identify barriers to eating intuitively and coping strategies.

Participants completed the 12 modules over a 14-week period and were reminded by email during the first 12 weeks when each new module became available. A new module became available only once the previous module was completed (as defined by clicking Done on the last page of the module). Participants could also receive a weekly notification via SMS that each new module was available. No recommendations were given regarding how frequently the intervention should be accessed. The “Mind, Body, Food” intervention was available until the final 3-month follow-up assessment, allowing participants to revisit and revise previous material.

**Figure 2:**
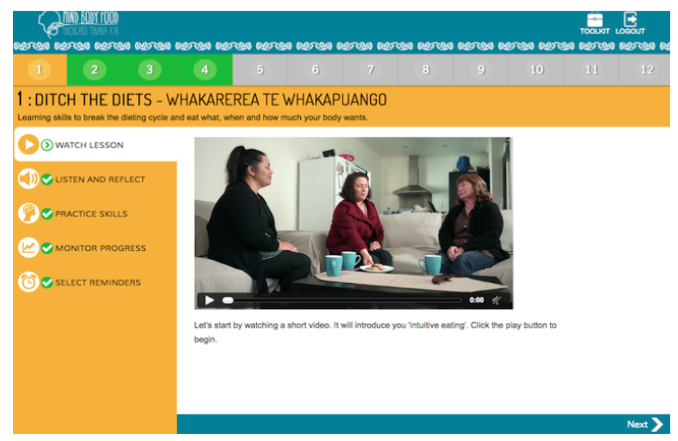
Video activity.

**Figure 3:**
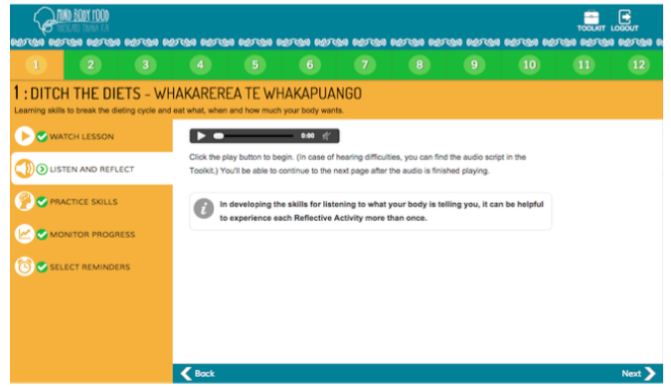
Audio activity.

**Figure 4:**
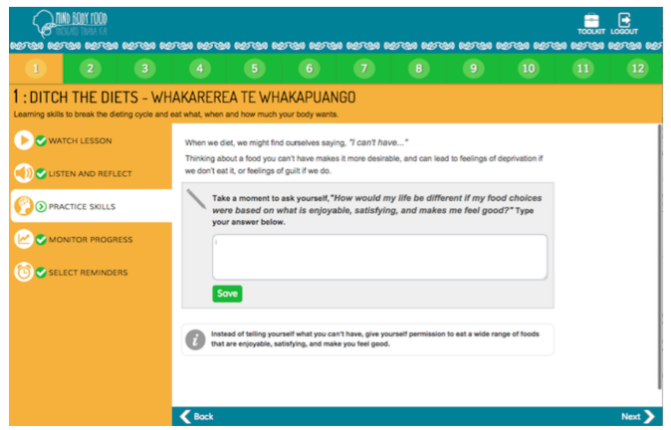
Typed activity.

**Figure 5:**
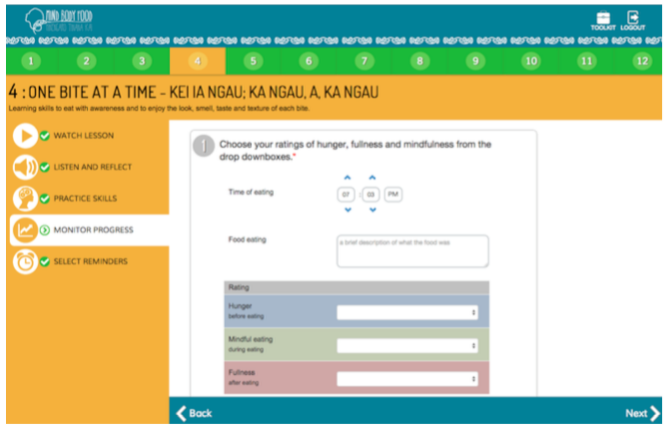
Eating Awareness Tracker (EAT) self-monitoring tool.

### Procedures

During the initial contact, a preliminary check of eligibility criteria was conducted. Preliminary screening criteria included premenopausal status, 40 to 50 years of age, and BMI greater than 26.5 kg/m^2^ based on self-reported height and weight. Women satisfying initial eligibility criteria were then emailed a Web-based screening questionnaire to evaluate exclusion criteria and Participant Information and Consent Form. Women who satisfied all inclusion and exclusion criteria were invited to participate in the study and were asked to complete the preintervention assessment by visiting the clinic located on campus. Women who did not meet eligibility criteria were offered a list of nondieting resources (eg, books, websites).

At the preintervention assessment, participants provided informed consent and upon enrollment in the study were assigned a username and passcode. At this time, they were given a brief tour of a module to familiarize them with the Web application. An intervention evaluation was completed online after the 12th module or during the postintervention clinic visit. At the final clinic visit, participants received a $20 grocery voucher as reimbursement for their costs in traveling to measurement sessions.

### Measures

#### Usage

Google Analytics [44] was used to record usage data for measuring the mean number of modules completed and mean session duration (one session was defined as a group of interactions with “Mind, Body, Food” until a log-out action or a period of 30 minutes of inactivity). User identification tracking (using email addresses at log-in) was enabled in Google Analytics to show data from individual users. The total number of modules completed was determined by the exit page (the last page a user viewed before exiting “Mind, Body, Food”) for each participant over the course of the study to identify the highest module number viewed. EAT usage was determined by the mean number of weeks participants saved at least one EAT entry to a secure online database over the 14-week trial. To determine if illness or technical difficulties affected EAT usage, all participants were asked in the postintervention questionnaire to report if for more than five days over the past 14 weeks their appetite had been affected or if their access to “Mind, Body, Food” had been limited.

#### Acceptability

A 32-item Web-delivered postintervention evaluation was developed to assess the acceptability of “Mind, Body, Food.” Acceptability questions were informed by the technology acceptance model [45] and focused on perceived usefulness (ie, the extent that using “Mind, Body, Food” enhances one’s ability to learn intuitive eating skills), ease of use, and user satisfaction. Questions such as “Overall, to what extent was ‘Mind, Body, Food’ useful to you?” were answered using 5-point Likert-type items (1—not useful to 5—extremely useful).

#### Participant Pre-, Post-, and 3-Month Follow-Up Characteristics

Paper-based questionnaires were administered during the preintervention assessment (62 items) and during both postintervention and 3-month follow-up assessments (56 items). All questionnaires were checked for completeness during the clinic visit, and participants were asked to answer any missed questions at that time.

The 23-item IES-2 [14] was used to measure women’s tendency to eat intuitively. The subscales measure eating for physical rather than emotional reasons, unconditional permission to eat, reliance on hunger and satiety cues to guide eating, and body-food choice congruence (eg, choosing to eat foods that provide energy and stamina). Statements (eg, “I trust my body to tell me when to eat”) were rated using subscales comprising 5-point Likert-type items (1—strongly disagree to 5—strongly agree). Providing evidence of its construct validity, the IES-2 total score and subscale scores have been positively related to body appreciation, self-esteem, and satisfaction with life and inversely related to eating disorder symptomology, poor interoceptive awareness, body surveillance, body shame, BMI, and internalization of media appearance ideals among women [14]. The IES-2 has also been found to yield internally consistent and stable scores with samples of women, providing evidence of reliability [14]. Preintervention, postintervention and 3-month follow-up IES-2 total score Cronbach alphas were .86, .90, and .91, respectively. Of the IES-2 subscales, all Cronbach alphas were .74 or higher (considered to be acceptable [46]) with three exceptions: preintervention Body-Food Choice Congruence subscale, postintervention and 3-month follow-up Unconditional Permission to Eat subscale Cronbach alphas were .61, .60, and .64, respectively.

Two questions were adapted from the Eating Disorder Examination-Screening Version (EDE-S) [47] to measure recent binge eating behavior. The first question asked, “Over the past 4 weeks (28 days), have there been any times when you have eaten what other people would regard as an unusually large amount of food?” to which participants were asked to respond Yes or No. Responses to this question were used to create a dichotomous variable (ie, binge eating vs no binge eating). The second question asked, “On how many days out of the last 28 have you had episodes like this when you may have also felt either unable to prevent them or unable to stop them once they had started?” Participants were asked to respond with the number of days. The EDE-S has high sensitivity (.90-.94) and specificity (.80-.96) in detecting eating disorders in community samples [47].

Height and weight were measured to calculate BMI for investigating preliminary effectiveness for weight gain prevention. A trained research assistant measured height to the nearest 0.1 centimeters using a stadiometer. Shoes were removed prior to the measurement. Weight was measured to the nearest 0.1 kilogram using a standard electronic scale while participants wore light clothing. Both height and weight were measured twice at each assessment and the mean values were used.

ACT-based processes (ie, psychological flexibility) were measured using the 7-item Acceptance and Action Questionnaire-II (AAQ-II) [48]. Items such as “My painful experiences and memories make it difficult for me to live a life that I would value” were answered using 7-point Likert-type items (1—never true to 7—always true). Higher scores reflected higher psychological *inflexibility*. The AAQ-II has good validity and reliability with various samples including college students and community-based adults [48]. Preintervention, postintervention, and 3-month follow-up AAQ-II scores had excellent Cronbach alphas (.91, .92, and .94, respectively).

Quality of life was measured by the Short Form 12-item Health Survey (SF-12v2) [49]. The SF-12v2 assesses eight domains of health, which are collapsed into two component summary measures of health: physical (incorporating Physical Functioning, Role-Physical, Bodily Pain, and General Health subscales) and mental (incorporating Vitality, Social Functioning, Role-Emotional, and Mental Health subscales) [50]. The component summaries have good reliability and validity among large community samples [51]. Preintervention, postintervention and 3-month follow-up SF-12v2 scores had acceptable Cronbach alphas (for physical summary measure: .79, .87, and .78, respectively; for mental summary measure: .84, .85, and .83, respectively).

Items assessing ethnicity, highest educational level, occupation status, and employment status were obtained from Statistics NZ’s Census 2006 [52]. Ethnicity data were used to assign responders to one of five categories in the following order: Māori, Pacific Islander, Asian, Other and NZ European. Occupational status was used to assess socioeconomic status using the NZ Socioeconomic Index (NZSEI-06) [53]. Classification codes were retrieved from an online searchable database provided by Statistics New Zealand to identify the appropriate NZSEI-06 score [53]. When participants recorded a partner’s occupation, the higher index score among the two was used to estimate household socioeconomic status. NZSEI-06 scores range from 10 to 90 with higher scores indicating higher socioeconomic status.

### Statistical Analysis

Mainly descriptive analyses were conducted due to the exploratory nature of the pilot study. Analyses were conducted using all available data. Means and standard deviations (SDs) for continuous variables and frequencies and percentages for categorical variables were calculated to describe the study sample. Analyses of differences between those completing and not completing the study and the within-group changes during the intervention were performed according to modified intention-to-treat principles, including all eligible women with available data but irrespective of their degree of participation with the intervention. Chi-squared tests and two sample *t* tests examined whether baseline characteristics predicted retention. Paired *t* and Wilcoxon matched-pairs signed-rank tests were performed to assess the overall significance of within-group changes over time among continuous variables, and McNemar tests were used in the same way for categorical variable comparisons. Spearman correlations were used to assess associations between changes from preintervention to postintervention in each of total IES-2 scores, the four IES subscale scores, BMI, and AAQ-II scores. Statistical analyses were performed using Stata 11.2 software (StataCorp LP) using 2-sided tests with the level of statistical significance set at *P*<.05.

## Results

### Recruitment and Retention

**Figure 6:**
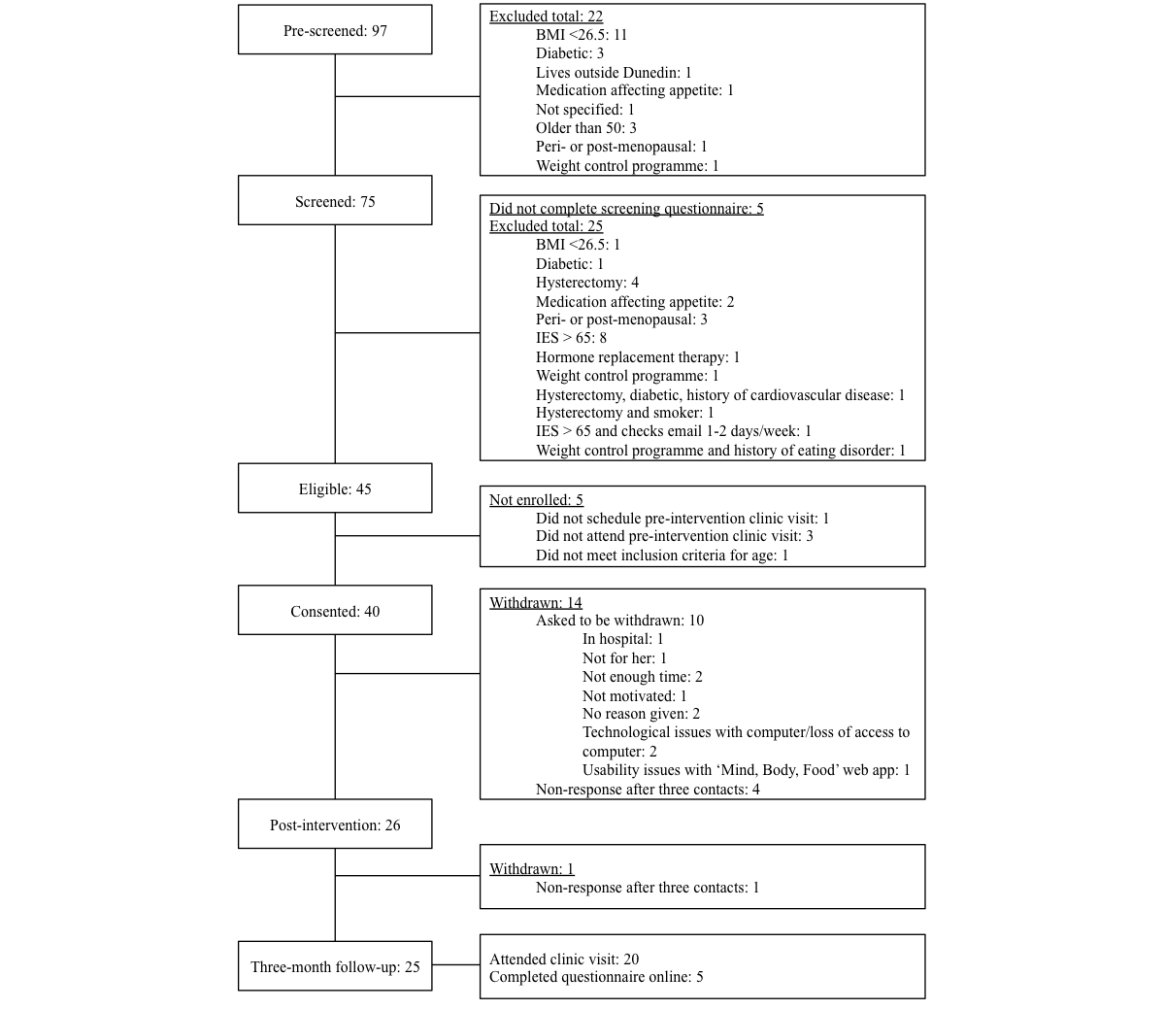
"Mind, Body, Food" study participant flow diagram.

During recruitment, of the 97 women who expressed interest in the study, 75 women met preliminary screening criteria and subsequently were invited to complete the online eligibility assessment. Five women did not complete the online eligibility assessment, and only 45 women met the study inclusion criteria (see Figure 6 for reasons women were excluded). A total of 40 women were enrolled in the study from August to September 2014. The postintervention retention rate was 65% (26/40) and 63% (25/40) at the 3-month follow-up. Although the attrition rate was similar to that predicted (30%), the sample size at follow-up was lower than had been aimed for due to the number of women not meeting eligibility criteria. Figure 6 presents the flow of participants through the study.

### Baseline Characteristics

Table 2 presents the sample’s demographic characteristics at baseline in comparison with corresponding NZ Census data. The sample was overrepresentative of NZ European women (30/37, 81.1% vs 69.8%) but similarly representative of Māori (4/37, 10.8% vs 11.7%) and Pacific (1/37, 2.7% vs 4.4%) women. University-educated women were overrepresented (21/37, 56.8% vs 17.7%). Women with NZSEI-06 (estimating socioeconomic status) scores in the lower two quartiles were underrepresented (10-33: 2/37, 5.4% vs 23.1%, 34-44: 3/37, 8.1% vs 29.4%). Half (18/36, 50.0%) of women reported dieting for 20 years or longer. There were no statistically significant differences in demographic characteristics or other measures such as intuitive eating, binge eating, general mental health, psychological inflexibility, and BMI between women who were retained and not retained at 3-month follow-up. 

**Table 2:** Baseline characteristics of “Mind, Body, Food” participants (n=40).

Characteristic		All participants	National data
Age, years, mean		44.8	—
IES-1^a^ summed total score [17], mean		53.4	69.4
**Ethnicity [54], %^b^**			
	New Zealand European	81.1	69.8
	Māori	10.8	11.7
	Pacific	2.7	4.4
	Other	5.4	4.6
**Highest level of education attained [54], %^b^**			
	Secondary school or less	16.2	59.1
	Technical/trade school or polytechnic	27.0	23.2
	University	56.8	17.7
**Employment [55], %^b^**			
	Employed	89.2	81.2-81.8
	Employed full time	59.5	—
	Employed part time	29.7	—
	Homemaker	5.4	—
	Other	5.4	—
**Socioeconomic status (NZSEI-06)^c^ [56], %^b^**			
	62-90 (higher socioeconomic status)	48.6	26.0
	45-61	37.8	21.5
	34-44	8.1	29.4
	10-33 (lower socioeconomic status)	5.4	23.1
BMI^d^ (kg/m^2^) [57], mean		32.92	27.8
**IES-2^e^ total, mean**		2.53	—
	UPE^f^, mean	3.06	—
	EPR^g^, mean	2.17	—
	RHS^h^, mean	2.33	—
	B-FCC^i^, mean	2.85	—
AAQ-II^j^, mean		22.01	—
PCS^k^, mean		50.01	—
MCS^l^, mean		46.32	—
**Binge eating, %^m^**			
	Yes	46.2	—
	No	53.8	—
**Dieting history, %^n^**			
	0-4 years	27.8	—
	5-19 years	22.2	—
	20+ years	50.0	—

^a^IES-1: Intuitive Eating Scale-1 (summed scores have potential range 21-105).

^b^Missing data, n=3.

^c^New Zealand Socioeconomic Index.

^d^BMI: body mass index.

^e^IES-2: Intuitive Eating Scale-2 (mean scores have potential range 1-5).

^f^UPE: Unconditional Permission to Eat subscale.

^g^EPR: Eating for Physical Rather than Emotional Reasons subscale.

^h^RHS: Reliance on Internal Hunger and Satiety cues.

^i^B-FCC: Body-Food Choice Congruence.

^j^AAQ-II: Acceptance and Action Questionnaire-II.

^k^PCS: Short Form 12-item (version 2) Health Survey Physical Component Summary.

^l^MCS: Short Form 12-item (version 2) Health Survey Mental Component Summary.

^m^Missing data, n=1.

^n^Missing data, n=4.

### Usage During the 14-week Intervention

Google Analytics recorded “Mind, Body, Food” online traffic for 37 participants for 92 days at the end of the 14-week trial. A programming delay prevented monitoring from the start of the study, which explains missing usage data for three women. Of the 37 participants for whom usage data were available, 12 (32%) completed all 12 modules, 11 (30%) completed 7 to 11 modules, 12 (32%) completed 1 to 6 modules, and two women (5%) completed no modules. The median number of modules completed was 7.5 (interquartile range [IQR] 2-12) modules. The median number of sessions during the monitored period was 7 (a minimum of 1 session to a maximum of 66 sessions). The median session duration during the monitored period was 12 minutes, 54 seconds (a minimum of 3 minutes, 58 seconds, to a maximum of 100 minutes, 8 seconds). The median number of EAT entries over the 14-week intervention was 7 (a minimum of 1 entry to a maximum of 314 entries). The median number of weeks that participants recorded eating experiences in the EAT was 3 (IQR 1-9) weeks. Of the 40 participants enrolled in the study, 4 women (10%) used the EAT for at least 12 weeks of their intervention period, 7 (18%) used the EAT for 7 to 11 weeks, 20 (50%) used the EAT for 1 to 6 weeks, and 9 (23%) did not use the EAT during their intervention period.

Eleven women had limited access to “Mind, Body, Food” for more than 5 days, predominantly due to being away from home (n=8) or technical issues (n=3). Technical issues that prevented module completion included attempting to access “Mind, Body, Food” from an incompatible browser (n=3), using incorrect login details (n=1), and other issues not described (n=1). Seven women reported appetite being affected for more than 5 days (eg, due to illness) during the 14-week intervention.

### Acceptability

A total of 26 women completed acceptability measures, and of these 12 had completed all modules, 10 completed 7 to 11 modules, and 4 completed 2 to 6 modules. Table 3 shows that participants’ overall impression of “Mind, Body, Food” was positive. Most participants liked “Mind, Body, Food” and found the intervention useful (20/26, 77%), easy to use (17/25, 68%), easy to understand (23/25, 92%), and would recommend the program to others (21/25, 84%). Nearly half (12/25, 48%) of participants reported that “Mind, Body, Food” made it easy to learn intuitive eating skills.

Of all module components, the videos were more frequently rated as being quite or extremely useful by participants compared to the audio and typed activities (Table 3). Five out of 24 women (21%) women rated the EAT self-monitoring tool as quite or extremely useful, 3 out of 19 women (16%) found email reminders to use the EAT useful, and 4 out of 12 women (33%) found SMS reminders to use the EAT useful (not shown). In regards to reminders sent to begin the next module, 17 out of 26 women (68%) rated the email reminders as quite or extremely useful. Of the women (9/24, 38%) who chose to receive SMS reminders to begin the next module, 6 (67%) rated the reminders as useful (not shown).

Of the 20 women who currently owned a smartphone, 16 (80%) reported that if it were available a mobile version of “Mind, Body, Food” would be quite or extremely useful to them.

**Table 3:** User experiences of “Mind, Body, Food” (n=26).

Acceptability measures			n (%)	Median
**Overall impression**				
	**Overall, to what extent did you like “Mind, Body, Food?”**			4
		Disliked very much	0 (0)	
		Disliked somewhat	0 (0)	
		Neither liked nor disliked	3 (12)	
		Liked somewhat	10 (40)	
		Liked very much	12 (48)	
		Missing	1	
	**Overall, to what extent was “Mind, Body, Food” useful to you?**			4
		Not useful	0 (0)	
		A little useful	2 (8)	
		Somewhat useful	4 (15)	
		Quite useful	9 (35)	
		Extremely useful	11 (42)	
	**Overall, to what extent did you find “Mind, Body, Food” easy to use?**			4
		Not at all easy	0 (0)	
		Not very easy	3 (12)	
		Somewhat easy	5 (20)	
		Very easy	12 (48)	
		Extremely easy	5 (20)	
		Missing	1	
	**Overall, to what extent did you find “Mind, Body, Food” content easy to understand?**			4
		Not at all easy	0 (0)	
		Not very easy	0 (0)	
		Somewhat easy	2 (8)	
		Very easy	15 (60)	
		Extremely easy	8 (32)	
		Missing	1	
	**Overall, to what extent did “Mind, Body, Food” make it easy for you to learn intuitive eating skills?**			3
		Not at all easy	0 (0)	
		Not very easy	2 (8)	
		Somewhat easy	11 (44)	
		Very easy	9 (36)	
		Extremely easy	3 (12)	
		Missing	1	
	**Would you recommend “Mind, Body, Food” to others?**			5
		No, definitely wouldn’t	0 (0)	
		No, probably wouldn’t	0 (0)	
		Unsure	4 (16)	
		Yes, probably would	8 (32)	
		Yes, definitely would	13 (52)	
		Missing	1	
**Usefulness of program features**				
	**How useful did you find the videos?**			4
		Not useful	0 (0)	
		A little useful	4 (16)	
		Somewhat useful	4 (16)	
		Quite useful	7 (28)	
		Extremely useful	10 (40)	
		Missing	1	
	**How useful did you find the audio activities?**			3
		Not useful	4 (16)	
		A little useful	5 (20)	
		Somewhat useful	7 (28)	
		Quite useful	7 (28)	
		Extremely useful	2 (8)	
		Missing	1	
	**How useful did you find the typed activities?**			3
		Not useful	4 (16)	
		A little useful	3 (12)	
		Somewhat useful	11 (44)	
		Quite useful	4 (16)	
		Extremely useful	3 (12)	
		Missing	1	
	**How useful did you find the E.A.T.?**			2
		Not useful	5 (21)	
		A little useful	9 (38)	
		Somewhat useful	5 (21)	
		Quite useful	3 (13)	
		Extremely useful	2 (8)	
		Missing	2	
	**How useful did you find the email reminders to begin the next module?**			3
		Not useful	2 (8)	
		A little useful	1 (4)	
		Somewhat useful	5 (20)	
		Quite useful	9 (36)	
		Extremely useful	8 (32)	
		Missing	1	

### Changes in Eating Behavior

The study was not designed to detect the effects of the intervention on the outcome measures (no control group was included), but within-group comparisons were performed as part of the feasibility component of the pilot study. From pre- to postintervention, there were statistically significant increases in IES-2 total scores and all four IES-2 subscale scores (Table 4). There was no evidence that these improvements diminished at the 3-month follow-up. Based on use of the dichotomous variable (ie, binge eating vs no binge eating), 7 out of 14 women who reported eating a significantly large amount of food (ie, binge eating) at the preintervention assessment reported no binge eating at the postintervention assessment. There were no women who transitioned from no binge eating at the preintervention assessment to binge eating at the postintervention assessment or at the 3-month follow-up; 4 women who reported binge eating at postintervention no longer reported binge eating at the 3-month follow-up. McNemar’s test revealed a significant decrease in the proportion of women binge eating from the pre- to postintervention visits (*P*=.01) and a significant decrease from postintervention to follow-up (*P*<.001). The median days women who were categorized as binge eaters reported binge eating at preintervention was 4.25 (IQR 2.5-10) days, at postintervention was 4 (IQR 2-8) days, and at the 3-month follow-up it was 2 (IQR 2-4) days. A Wilcoxon matched-pairs signed-rank test was used to examine changes in the reported number of days women were unable to prevent or stop episodes of eating an unusually large amount of food and revealed a significant decrease from pre- to postintervention (*P*=.03) and no evidence of a change from postintervention to follow-up (*P*=.57).

### Psychological Inflexibility, Quality of Life, and BMI

Psychological inflexibility decreased significantly from pre- to postintervention (mean change −4.23 [SD 7.13], *P*=.006), and there was a tendency for a further decrease in psychological inflexibility from postintervention to 3-month-follow-up (mean change −2.60 [SD 6.58], *P*=.06) (Table 4). There were no significant changes in general physical health from pre- to postintervention (pre- to postintervention PCS scores: mean change 0.29 [SD 9.35], *P*=.88; postintervention to 3-month follow-up PCS scores: mean change 1.26 [SD 10.46], *P*=.55). However, general mental health improved significantly from pre- to postintervention with a further improvement postintervention to 3-month follow-up (mean change 5.07 [SD 6.31], *P*<.001; mean change 3.45 [SD 7.71], *P*=.03, respectively). There were no statistically significant changes in BMI from pre- to postintervention or postintervention to 3-month follow-up (mean change −0.13 [SD 0.88], *P*=.46 and mean change −0.01 [SD 0.57], *P*=.93, respectively).

### Associations Between Changes in Intuitive Eating With BMI and Psychological Inflexibility

Spearman correlations showed a statistically significant inverse relationship between pre- to postintervention change in total intuitive eating scores and pre- to postintervention change in BMI (*r*_s_=−.43, *P*=.03). Thus, greater decreases in BMI were associated with greater increases in intuitive eating. However, when relationships with individual subscales were examined, a significant association was found for only one subscale: greater decreases in BMI were associated with greater increases in eating for physical rather than emotional reasons (*r*_s_=−.56, *P*=.003). There was also a statistically significant inverse relationship between change in psychological inflexibility scores and change in intuitive eating scores (*r*_s_=−.52, *P*=.006); that is, greater improvements in psychological flexibility were associated with greater increases in total intuitive eating scores. Furthermore, larger improvements in psychological flexibility were associated with greater increases in two subscale scores: eating for physical rather than emotional reasons (*r*_s_=−.41, *P*=.04) and reliance on hunger and satiety cues (*r*_s_=−.62, *P*<.001). A greater reduction in psychological inflexibility scores was also associated with a greater decrease in BMI (*r*=.41, *P*=.04).

**Table 4:** Changes from pre- (n=26) to postintervention (n=26) and from postintervention to 3-month follow-up (n=25).

Characteristic		Mean (SD)	Change (SD)^a^	95% CI	*P* value^b^
IES-2^c^ total					
	Preintervention	2.54 (0.58)	—	—	—
	Postintervention	3.45 (.55)	0.94 (0.67)	0.67, 1.21	<.001
	3-month follow-up	3.53 (.61)	0.08 (0.55)	−0.15, 0.31	.47
UPE^d^					
	Preintervention	3.07 (0.73)	—	—	—
	Postintervention	3.56 (.54)	0.52 (0.87)	0.17, 0.87	.01
	3-month follow-up	3.47 (.58)	−0.09 (0.64)	−0.36, 0.17	.76
EPR^e^					
	Preintervention	2.19 (0.96)	—	—	—
	Postintervention	3.21 (.89)	1.08 (0.96)	0.69, 1.46	<.001
	3-month follow-up	3.49 (.88)	0.28 (0.89)	−0.09, 0.65	.13
RHS^f^					
	Preintervention	2.33 (0.77)	—	—	—
	Postintervention	3.58 (.76)	1.28 (0.98)	0.89, 1.68	<.001
	3-month follow-up	3.55 (.81)	−0.03 (0.68)	−0.31, 0.25	.81
B-FCC^g^					
	Preintervention	2.87 (0.61)	—	—	—
	Postintervention	3.59 (.81)	0.74 (0.61)	0.50, 1.00	<.001
	3-month follow-up	3.72 (.72)	0.13 (0.87)	−0.23, 0.49	.45
BMI^h^ (kg/m^2^)					
	Preintervention	32.93 (4.85)	—	—	—
	Postintervention	32.81 (5.31)	−0.13 (0.88)	−0.48, 0.23	.46
	3-month follow-up^i^	32.80 (5.29)	−0.01 (0.57)	−0.28, 0.26	.93
AAQ-II^j^					
	Preintervention	22.46 (8.67)	—	—	—
	Postintervention	18.23 (8.14)	−4.23 (7.13)	−7.11, -1.35	.006
	3-month follow-up	16.08 (7.73)	−2.60 (6.58)	−5.32, 0.16	.06
SF-12v2 PCS^k^					
	Preintervention	50.46 (7.83)	—	—	—
	Postintervention	50.87 (11.02)	0.29 (9.35)	−3.49, 4.06	.88
	3-month follow-up	52.13 (6.78)	1.26 (10.46)	−3.06, 5.58	.55
SF-12v2 MCS^l^					
	Preintervention	45.63 (6.31)	—	—	—
	Postintervention	50.24 (7.41)	5.07 (6.31)	2.52, 7.61	<.001
	3-month follow-up	53.70 (4.99)	3.45 (7.71)	0.27, 6.63	.03

^a^Changes from pre- to postintervention and postintervention to 3-month follow-up.

^b^Paired *t* tests were used to compare IES-2 scores, BMI, AAQ-II scores, and SF-12v2 scores.

^c^IES-2: Intuitive Eating Scale-2.

^d^UPE: Unconditional Permission to Eat subscale.

^e^EPR: Eating for Physical Rather than Emotional Reasons subscale.

^f^RHS: Reliance on Internal Hunger and Satiety cues.

^g^B-FCC: Body-Food Choice Congruence.

^h^BMI: body mass index.

^i^BMI change from postintervention to 3-month follow-up (n=20).

^j^AAQ-II: Acceptance and Action Questionnaire-II.

^k^PCS: Short Form 12-item (version 2) Health Survey Physical Component Summary.

^l^MCS: Short Form 12-item (version 2) Health Survey Mental Component Summary.

## Discussion

### Principal Findings

This pilot study is the first evaluation of an online intuitive eating intervention and demonstrated that middle-aged overweight women perceived the intervention to be an acceptable and useful way to learn intuitive eating skills. Among those women who completed all assessments, both psychological flexibility and all aspects of intuitive eating improved at the end of the intervention and were sustained at the 3-month follow-up. Binge eating and general mental health improved from preintervention to postintervention and there were further improvements in these outcomes at the 3-month follow-up. Results are consistent with the hypothesis that improvements in psychological flexibility may be linked with eating more intuitively.

To our knowledge, only one other published intuitive eating intervention (a face-to-face intervention) [58] has shown improvements in all aspects of intuitive eating (ie, unconditional permission to eat when hungry and what food is desired, eating for physical reasons, trusting bodily cues to determine when and how much to eat), as has been demonstrated in this pilot study. An innovative feature and major strength of the present study is the integration of intuitive eating with ACT skills. Many ACT skills are designed to build awareness and acceptance of internal experiences, which may in turn foster the ability to tune into and trust the body’s hunger and satiety cues and not react to emotions and cravings by eating. ACT’s focus on values- guided behavior may strengthen women’s commitment to eating intuitively by helping them to clarify the value of shifting to eating behavior that is enjoyable, sustainable, empowering, and health-focused rather than weight-focused. The ACT elements of the intervention may have also improved nonjudgment towards thoughts about food or feelings about one’s body and reduced reactivity towards experiences (ie, responding to internal and external experiences in deliberate and meaningful ways). Furthermore, the significant increase in psychological flexibility suggests the ACT strategies were active in the intervention. This study demonstrates support for and justifies further exploration of our innovative approach of integrating intuitive eating with ACT.

The significant reduction in binge eating behavior in this study is consistent with previous intuitive eating interventions [19] and ACT-based interventions [25,27]. Those who learn intuitive eating appear to be able to reduce the loss of control that often follows self-imposed restriction [18], and this ability may be enhanced by improved coping with emotional distress through development of ACT skills.

Improvements in women’s general mental health may be explained by the ACT-based skills taught in “Mind, Body, Food;” however, intuitive eating interventions which do not specifically incorporate ACT skills have also been reported to improve psychological well-being [18]. Enhanced awareness and being less judgmental may have benefited women in areas of life beyond their eating experiences, allowing them to respond to situations in more personally meaningful ways. Physical activity was not addressed until the tenth module, which was viewed by a minority of participants, and this may contribute to the absence of changes in general physical health.

It is encouraging that numerous improvements occurred in spite of only 30% of the sample completing all 12 modules. Low completion rates are typical of Web-based interventions [43,59,60]. A systematic review of trials of Web-based health promotion interventions reported that on average 50% (minimum 1%; maximum 93%) of study participants completed all intervention modules (with interventions typically 10 modules long and meant to be used once a week) [60]. However, in this review, 76% of the interventions included interaction of the participant with a counselor, a factor which predicted significantly better adherence [60] but was lacking in our “Mind, Body, Food” intervention. A recent Web-based weight gain prevention intervention more similar to ours has reported the percentage of women completing all modules to be lower than in our study [61]. Low completion rates in such interventions, including our own, may reflect waning interest, perceiving the early modules of the intervention as sufficiently useful for learning new skills and thereby feeling it unnecessary to complete the rest, or a lack of social support [62]. Further investigation could assist in determining how many and which combination of modules are associated with optimal improvement in intuitive eating prior to a future RCT. Another contributor to noncompletion may be the desire for weight loss rather than a shift in focus towards long-term healthier lifestyle behaviors [62], particularly among women starting at a higher BMI [63] or among those with high body dissatisfaction.

Low use of the EAT and the importance of self-monitoring to successful behavior change [64-66] suggest that simplification and improvement of the self-monitoring tool is needed. In response to feedback from target users during intervention development, women had to choose to receive reminders to use the EAT rather than receiving automatic reminders, and this may also have contributed to low usage. However, the reported improvements in intuitive eating and binge eating may suggest that for some women, a self-monitoring tool may not have been essential for learning intuitive eating.

### Strengths and Limitations

The study strengths include an intervention that integrates two empirically tested approaches to eating behavior change (intuitive eating and ACT), use of an intervention design that was informed by input from end-users and underwent rigorous pretesting with women in the target audience prior to the pilot study; objective measures of BMI and intervention usage; validated measures of intuitive eating, binge eating, psychological flexibility, and quality of life; and a 3-month follow-up. The most significant limitations of the study were the lack of a comparison group, small sample size, analyses conducted on the subset of participants who were exposed to the intervention and had not dropped out for any reason, and delayed monitoring via Google Analytics; however, the pilot study was undertaken to inform improvements to the intervention prior to proceeding to an RCT incorporating a control group. The pilot study shared the high attrition common to many Web-based interventions and results were based on a small group completing the study. The high rate of attrition observed during the study (35% were lost by postintervention and 37% by 3-month follow-up) and the expectation that missing data from health-focused studies involving overweight participants will generally be informative means that we cannot be certain that those dropping out did not experience negative outcomes that could attenuate or even negate the positive findings reported here. Prior to a future randomized trial, further research is needed to determine ways to increase engagement and improve program completion. This may be achieved by making the tool smartphone compatible since accessing the program on the go is likely to be helpful to women in applying the skills in real-world settings. A reduction in the number of modules may also be useful. The sample was overrepresentative of highly educated women, which has been noted in several studies of Web-based weight management interventions [67-69], and results may not generalize to less educated women. The positive self-reported outcomes may reflect regression to the mean, repeated administration of the questionnaires, or social desirability bias.

### Conclusion

To our knowledge, “Mind, Body, Food” is the first intervention to combine teaching intuitive eating skills and ACT-based strategies for behavior change. It is also the first Web-based intuitive eating intervention. The “Mind, Body, Food” intervention was generally acceptable to the target audience, and the pilot study showed improvements in intuitive eating, binge eating behavior, psychological flexibility and general mental health, in addition to weight maintenance. Given the 3-month follow-up period and lack of a control group, it is important that an improved version of the intervention is evaluated in an RCT to investigate its longer-term effectiveness. The RCT will also examine the hypothesized mechanism that greater psychological flexibility leads to more intuitive eating, which leads to prevention of weight gain.

## Abbreviations

AAQ-II: Acceptance and Action Questionnaire
ACT: acceptance and commitment therapy
B-FCC: body-food choice congruence
BMI: body mass index
EAT: Eating Awareness Tracker
EDE-S: Eating Disorder Examination-Screening Version
EPR: eating for physical rather than emotional reasons
IES: Intuitive Eating Scale
IES-2: Intuitive Eating Scale-2
IQR: interquartile range
MCS: Mental Component Summary
NZSEI-06: New Zealand Socioeconomic Index
PCS: Physical Component Summary
RCT: randomized controlled trial
RHS: reliance on internal hunger and satiety cues
SD: standard deviation
SES: socioeconomic status
SF-12v2: Short Form 12-item (version 2) Health Survey
SMS: short message service
UPE: unconditional permission to eat
